# YgaE Regulates Out Membrane Proteins in *Salmonella enterica* Serovar Typhi under Hyperosmotic Stress

**DOI:** 10.1155/2014/374276

**Published:** 2014-01-23

**Authors:** Min Wang, Ping Feng, Xun Chen, Haifang Zhang, Bin Ni, Xiaofang Xie, Hong Du

**Affiliations:** ^1^Clinical Laboratory, The Second Affiliated Hospital of Soochow University, Suzhou 215004, China; ^2^Clinical Laboratory Center, Xiyuan Hospital, China Academy of Chinese Medical Sciences, Beijing 100091, China; ^3^Department of Biochemistry and Molecular Biology, School of Medical Technology, Jiangsu University, Zhenjiang 212013, China

## Abstract

*Salmonella enterica *serovar Typhi (*S*. Typhi) is a human-specific pathogen that causes typhoid fever. In this study, we constructed Δ*ygaE *mutant and a microarray was performed to investigate the role of *ygaE *in regulation of gene expression changes in response to hyperosmotic stress in *S*. Typhi. qRT-PCR was performed to validate the microarray results. Our data indicated that *ygaE *was the repressor of *gab* operon in *S*. Typhi as in *Escherichia coli* (*E. coli*), though the sequence of *ygaE* is totally different from *gabC *(formerly *ygaE*) in *E. coli*. OmpF, OmpC, and OmpA are the most abundant out membrane proteins in *S*. Typhi. Here we report that YgaE is a repressor of both OmpF and OmpC at the early stage of hyperosmotic stress. Two-dimensional electrophoresis was applied to analyze proteomics of total proteins in wild-type strain and Δ*ygaE *strain and we found that YgaE represses the expression of OmpA at the late stage of hyperosmotic stress. Altogether, our results implied that YgaE regulates out membrane proteins in a time-dependent manner under hyperosmotic stress in *S*. Typhi.

## 1. Introduction

*S*. Typhi is a human-specific pathogen, which produces typhoid fever. Once ingested through contaminated water or food, *S*. Typhi invades intestinal epithelial cells and can enter the host bloodstream and disseminate to deep organs. Lacking of standard water supply and sanitation, typhoid remains a major health problem in developing world [1–3]. In contaminated water or food the external osmolarity is in the order of 50 mM NaCl; however, in the lumen of the host intestine *S*. Typhi cells are exposed to a significant increase in osmolarity: 300 mM NaCl in the small intestine [4]. Considering the significant morbidity and mortality associated with this disease [5], it is important to understand the gene regulation mechanisms in *S*. Typhi in response to hyperosmotic environments.

The outer membrane (OM) of Gram-negative bacteria constitutes the first permeability barrier that protects the cells against environmental stresses including chemical and biological attacks [6]. Simultaneously, it allows the selective uptake of essential nutrients and the secretion of metabolic waste products. The OM is a sophisticated organization of lipid and protein components. The outer membrane proteins (OMPs), called porins, are characterized by a *β*-barrel structure and form water-filled channels for the passage of a large variety of hydrophilic molecules [7–9].

In *E. coli*, the two general porins OmpF and OmpC are among the most abundant OMPs (about 10^5^ copies per cell) and serve as general pathways for the influx of small molecules (e.g., molecular weight under 600). They consist of three 16-stranded *β*-barrels, each of which forms a channel that is restricted in the middle due to the inward folding of a loop (loop L3) [10]. The expression of *E. coli* porins has been extensively studied. The OmpF-OmpC balance is highly regulated by different genetic control systems. Changes in osmolarity profoundly affect expression of OmpF and OmpC. OmpC is preferentially expressed in high osmolarity, whereas OmpF expression is favored in low osmolarity [11]. Other factors, including local anesthetics [12], pH [13], and nutrition limitation [14] also influence *ompF *and *ompC *transcription in an EnvZ/OmpR-dependent manner. Noteworthy, growth conditions where nutrient levels are high, such as in mammal intestinal tracts, favor the expression of OmpC, which has a smaller channel than OmpF, thus limiting the influx of large and charged molecules such as bile salts and antibiotics. Conversely, OmpF will be the major porin under ex vivo growth conditions with nutritional deficiency, as its larger pore will allow efficient influx of nutrients [15]. In contrast, expression of OmpC in *S*. Typhi is not influenced by osmolarity, while OmpF is regulated as in *E. coli* [16].

OmpA is another abundant OMP. It is monomeric, and it is unusual in that it can exist in two different conformations [17]. A minor form of the protein, with an unknown number of transmembrane strands, can function as a porin, but the major, nonporin form has only eight transmembrane strands, and the periplasmic domain of this form performs a largely structural role [18]. The function of OmpA is thought to contribute to the structural integrity of the outer membrane along with murein lipoprotein [19] and peptidoglycan associated lipoprotein [20].

In *E. coli*, *gabC* (formerly *ygaE*) was reported to belong to the *gabDTPC* operon, and *gabC* is the repressor of the operon [21]. The function of *gab* operon is mainly revolved in *γ*-aminobutyrate (GABA) catabolism but does not contribute to the catabolism of any other nitrogen source [21]. However, the function of *ygaE* in *S*. Typhi has not been extensively studied. In this work, we showed that *ygaE* regulates out membrane proteins OmpF/OmpC at the early stage of hyperosmotic stress and OmpA at the late stage of hyperosmotic stress in *S*. Typhi.

## 2. Materials and Methods

### 2.1. Bacterial Strains and Conditions of Culture

The bacterial strains and plasmids used in this study are listed in Table 1. Bacteria were grown in LB broth at 37°C with shaking (250 rpm). As for antibiotic sensitivity assay, Muller Hinton agar was used. For low environmental osmolarity, the growth medium contained a final concentration of 50 mM NaCl. For hyperosmotic environment, Nacl was added into the medium at a final concentration of 300 mM. The complemented strains were induced by L-arabinose (0.2% wt/vol). When appropriate, ampicillin was added to the medium at a final concentration of 100 *μ*g/mL.

### 2.2. Construction of the *ygaE* Deletion Mutant Strain

The *ygaE* deletion mutant (Δ*ygaE*) was prepared by homologous recombination according to a previously described method [24] with a *ygaE*-deletion suicide plasmid lacking 327 bp of the *ygaE* gene. The specific primers used for deletion of *ygaE* were listed in Table 2. The mutant strain was selected by PCR as described previously [23], verified by sequencing, and designated Δ*ygaE*.

### 2.3. Complement of *ygaE* in the Δ*ygaE* Mutant Strain

Specific primers *ygaE*-PA and *ygaE*-PB (Table 2) were designed to amplify a *ygaE* promoterless DNA fragment with *pfu* DNA polymerase (Takara). Nco I and Sal I sites were added to the 5′-termini of primers *ygaE*-PA and *ygaE*-PB, respectively. The amplicon was digested by Nco I and Sal I and inserted into the expression vector pBAD/gIII (Invitrogen), which was predigested with the same restriction enzymes, to form the recombinant plasmid pBAD*ygaE*. The positive plasmid was verified by sequence analysis. Δ*ygaE *was transformed with pBAD*ygaE* and designated as Δ*ygaE* (pBAD*ygaE*). As control, Δ*ygaE *was transformed with pBAD and designated as Δ*ygaE* (pBAD). Expression of *ygaE* in Δ*ygaE* (pBAD*ygaE*) was induced by L-arabinose (0.2% wt/vol).

### 2.4. RNA Extraction and Transcriptional Profiling by Genomic DNA

Wild-type and Δ*ygaE *strains were cultured overnight at 37°C with shaking (250 rpm) in LB broth (with final concentration of 50 mM). After dilution into fresh medium, cultures were incubated to exponential growth (OD 0.5 at 600 nm). To induce hyperosmotic stress, NaCl was added to a final concentration of 300 mM and bacteria were incubated with shaking for a further 30 min at 37°C. Bacteria were collected by centrifugation and total RNA was extracted using an RNeasy kit (minicolumn, Qiagen, Germany) according to the manufacturer's instructions. The quality and quantity of the extracted RNA were determined by agarose gel electrophoresis and analysis with a ND-1000 spectrophotometer (NanoDrop Technologies). Extracted RNA was treated with 1 U of RNase-free DNase I (TaKaRa) at 37°C for 10 min to remove traces of DNA and then incubated at 85°C for 15 min to inactivate the DNase. cDNA probes were synthesized using 20 *μ*g of RNA. A genomic DNA microarray designed for *S*. Typhi was used in this study and fluorescence labeling of cDNA probes, hybridization, and microarray scanning were performed as described previously [25].

### 2.5. Data Analysis

GENEPIX PRO 6.0 (Molecular Devices) was used for signal quantification. The densitometric values of the spots with DNA sequences representing open reading frames (ORFs) were normalized to the average overall intensity of the slide in global normalization mode. Data were exported into an Excel (Microsoft Corporation) spreadsheet for subsequent analysis as described previously [25] with minor modifications. In brief, the two-channel fluorescent intensity ratios were calculated for each individual spot on each slide; the average intensity ratio of the same gene from different slides was taken as the mean change in gene expression level. This was expressed as log2 (ratio) and entered as one data point in the gene expression profile plot view. Only genes that displayed at least eight valid values in 12 replicate analyses were subject to further analysis.

### 2.6. Quantitative Real-Time RT-PCR (qRT-PCR) Assay

Total RNA extracted after 30 min of hyperosmotic stress as above was subjected to qRT-PCR as described previously [26]. The PCR primers used for qRT-PCR are listed in Table 2. Each experiment was performed with three RNA samples from three independent experiments. Student's *t-*test was used for the statistical analysis. Differences were considered statistically significant when *P* was <0.05 in all cases.

### 2.7. Measurement of Bacterial Growth

Wild-type and *ygaE* mutant were overnight cultured; then 200 *μ*L aliquots of the culture were diluted to 20 mL fresh LB medium (with NaCl concentration of 300 mM) and incubated at 37°C with shaking (250 rpm). The growth was measured every two hours using a BioPhotometer (Eppendorf). The measurement was performed three times. Student's *t*-test was used for the statistical analysis. Differences were considered statistically significant when *P* was <0.05.

### 2.8. Measurement of Antibiotic Susceptibility

The antibiotic susceptibility testing was done by using the modified Kirby-Bauer disk diffusion method on Muller Hinton agar (OXOID) with a final NaCl concentration of 300 mM. The antibiotic disks which were used in this study were cefotaxime (CTX), ampicillin (AMP), piperacillin (PRL), ceftazidime (CAZ), compound sulfamethoxazole (SXT), and chloramphenicol (C). The zone size around each antimicrobial disk was measured. The experiment was performed three times. Student's *t*-test was used for the statistical analysis. Differences were considered statistically significant when *P* was <0.05.

### 2.9. Protein Extraction

Wild-type and Δ*ygaE *strains were cultured overnight at 37°C with shaking (250 rpm) in LB broth (with final concentration of 50 mM). After dilution into fresh medium, cultures were incubated to exponential growth (OD 0.5 at 600 nm). To induce hyperosmotic stress, NaCl was added to a final concentration of 300 mM and bacteria were incubated with shaking for a further 120 min at 37°C. Bacteria were collected by centrifugation. The cell pellets were washed twice with ice-cold PBS, resuspended in PBS, and sonicated for 10 sec with a Sonoplus sonicator (Bandelin electronic, Germany). The cells were collected by centrifugation at 5,000 g for 20 min. The resulting cell pellet was resuspended in sample lysis solution, which was composed of 7 M urea, 2 M thiourea containing 4% (w/v) 3-[(3-cholamidopropyl) dimethylammonio] -1-propanesulfonate (CHAPS), 1% (w/v) dithiothreitol (DTT) 2% (v/v) pharmalyte, and 1 mM benzamidine. Proteins were extracted for 1 h at room temperature with vortexing. After centrifugation at 15,000 g for 1h at 15°C, the insoluble material was discarded, and the soluble fraction was harvested and used for 2-DE.

### 2.10. Two-Dimensional Electrophoresis (2-DE)

The total proteins were dissolved in IPG rehydration/sample buffer (8 M urea, 2% CHAPS, 50 mM DTT, 0.2% Bio-Lyte 4/7 ampholyte, 0.001% Bromophenol Blue; Bio-Rad) and centrifuged at 12,000 g for 15 min at room temperature to remove nondissolved materials. The protein content was determined using the PlusOne 2D Quant Kit (Amersham Pharmacia Biotech). A 7 cm Immobiline DryStrip (IPG, Immobilized pH Gradient, pH range 4–7; Bio-Rad) was rehydrated at 50 V for 12 h, in IPG rehydration/sample buffer containing 150 mg of the protein sample in a total volume of 125 mL. Isoelectric focusing was performed using a Bio-Rad PROTEAN IEF cell (Bio-Rad) and focusing was conducted by stepwise increase of the voltage as follows: 250 V for 0.5 h, 500 V for 0.5 h, 4000 V for 3 h, and 4000 V until 25,000 Vh. The temperature was maintained at 20°C. After IEF separation, each IPG strip was washed in 3 mL of equilibration buffer 1 (75 mMTris—HCl [pH 8.8], 6 Murea, 2% SDS, 29.3% [v/v] glycerol, 1% DTT) for 15 min and in 3 mL of equilibration buffer 2 (75 mM Tris—HCl [pH 8.8], 6 M urea, 2% SDS, 29.3% [v/v] glycerol, 2.5% iodoacetamide) for an additional 15 min·IPG strips were then placed over a 12% resolving polyacrylamide gel and electrophoresis was performed in two steps at 10°C : 15 mA/gel for 30 min and 30 mA/gel until the tracking dye reached the bottom of the gels. All gels were stained with colloidal Coomassie Brilliant Blue G-250 (CBB). Gel evaluation and data analysis were carried out using the PDQuest v 7.3 program (Bio-Rad). Three replicates were run for the sample.

### 2.11. Mass Spectrometry Analysis of Protein Spots and Database Searches

Spots unique to both strains were excised from the 2-DE gels and sent to Shanghai GeneCore BioTechnologies Co., Ltd for tryptic in-gel digestion, MALDI-TOF-MS, and MALDI-TOF/TOF-MS Data from MALDI-TOF-MS and MALDI-TOF/TOF-MS acquisitions were used in a combined search against the NCBInr protein database using MASCOT (Matrix Science) with the parameter sets of trypsin digestion, one max missed cleavages, variable modification of oxidation (M), and peptide mass tolerance for monoisotopic data of 100 ppm. Originally, the MASCOT server was used against the NCBI for peptide mass fingerprinting (PMF). The criteria used to accept protein identifications were based on PMF data, including the extent of sequence coverage, number of peptides matched, and score of probability. Protein identification was assigned when the following criteria were met: at least four matching peptides and sequence coverage greater than 15% [27, 28]. The identification of protein spots with a lower Mascot Score required further confirmation by MALDI-TOF/TOF-MS.

## 3. Results and Discussion

### 3.1. YgaE Represses the Expression of *gab* Operon under Hyperosmotic Stress

In our previous work, we investigated the global transcriptional profiles of *S*. Typhi Δ*rpoE*, Δ*rpoS,* and Δ*rpoE*/Δ*rpoS* strains after 30 min of hyperosmotic stress by *Salmonella* genomic DNA microarray. The results of microarray indicated that the expression level of *ygaE* is dramatically reduced in Δ*rpoE*/Δ*rpoS* strain [29], while no apparent downregulation is observed in either Δ*rpoE* or Δ*rpoS* strain (data not shown). We speculated that *ygaE*, coregulated by RpoE and RpoS, is required for survival under extreme stresses of *S*. Typhi. To investigate the role of *ygaE* in the regulation of gene expression changes in response to hyperosmotic stress in *S*. Typhi, the *ygaE* mutant was constructed by homologous recombination mediated by suicide plasmid. Then, a genomic DNA microarray was performed to analyze the global transcriptional profiles of wild-type and Δ*ygaE* strains after 30 min of exposure to hyperosmotic stress.

The microarray results exhibited that, compared to wild-type strain, the expression, of *ygaF*, *gabD*, and *gabT* were obviously upregulated in Δ*ygaE* strain (Table 3) after exposure to hyperosmotic stress 30 min, which indicated that *ygaE* is the repressor of these genes at the early stage of hyperosmotic stress.

In *E. coli*, it was reported that there is an operon structure for the *gab* genes and that four genes form the *gabDTPC* operon [21]. The evidence that the first three genes are members of the *gab* operon is unambiguous [21]. Though the evidence that *gabC* (*ygaE*) is also a member of this operon is reasonably convincing, there still exist disputes. Firstly, it is unusual for a repressor to be encoded within the operon that it regulates. Next, a four-gene *gab* operon transcript was failed to be found [21]. *ygaF*, the gene preceding *gabD*, is not included in the *gab* operon in *E.coli *for several strong evidence [21].

**Figure 1:**
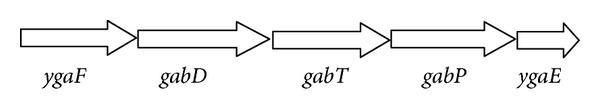
The *gab* operon structure in *S*. Typhi. The arrowhead represents the length of the gene; the arrowhead of *ygaE* corresponds to 0.678 kb.

In *E. coli*, *gabT* codes for a GABA transaminase that generates succinic semialdehyde. *gabD* specifies an NADP-dependent succinic semialdehyde dehydrogenase, which oxidizes succinic semialdehyde to succinate [30]. GabC does not obviously respond to a specific inducer. GabC is in the FadR subfamily of the GntR family of transcriptional regulators [31]. In *S*. Typhi, *gabD* encodes for a succinate-semialdehyde dehydrogenase, *gabT* encodes for a 4-aminobutyrate aminotransferase, and *ygaF* is a putative GAB DTP gene cluster repressor (Table 3). We compared the sequences of *gab* operon of *S*. Typhi to that of *E. coli* and found they are about 80% homologous. However, despite the same regulation pattern to *gab* operon, the sequence of *ygaE* in *S*. Typhi is totally different from *gabC* in *E. coli, *which also indicates that *ygaE* in *S*. Typhi may play other roles that is not found in *E. coli*. The gene organization of *gab* operon in *S*. Typhi was shown in Figure 1. Our microarray results suggested that YgaE can response to osmotic pressure in early stage to repress the expression of *gab* operon. However, the concrete regulation mechanism, whether *ygaF* is included in the *gab* operon and the functions of *gab* operon in *S*. Typhi, still needs further study.

The expression of *gabP* was failed to be detected both in wild-type and Δ*ygaE* strains due to the lack of *gabP* probe on the microarray used in this study.

### 3.2. YgaE Represses the Expression of *ompF/ompC* at the Early Stage of Hyperosmotic Stress

To conquer the often hostile environments they face, the bacteria have evolved a sophisticated cell envelope. The cell envelope of bacteria not only protects them from hazards but also provides them with channels for nutrients from the outside and wastes from the inside. In the envelope, there are three major compartments: the out membrane (OM), the periplasm, and the inner membrane (IM). The OM is a distinguishing feature of Gram-negative bacteria; Gram-positive bacteria lack this organelle [18]. The proteins of OM can be divided into two classes: lipoproteins and *β*-barrel proteins; the latter is the so-called out membrane proteins (OMPs). OmpF and OmpC are two abundant OMPs, which together are present at approximately 10^5^ copies per cell and they serve as general pathways for the influx of small molecules (e.g., molecular weight under 600) [32]. In *E. coli*, *ompC* is preferentially expressed under conditions of high osmolarity [8]. However, *ompC* is regulated differently in *S*. Typhi, in which *ompC* is expressed constitutively under conditions of high and low osmolarity [33, 34], while *ompF* is preferentially expressed under low osmolarity as in *E. coli* [16].

**Figure 2:**
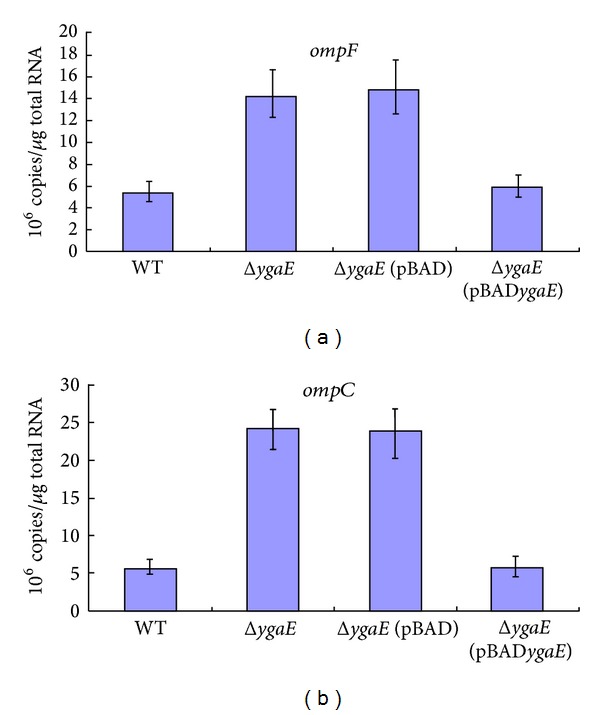
qRT-PCR was performed to detect expression of out membrane proteins in wild-type strain(WT), Δ*ygaE, *Δ*ygaE*(pBAD), and Δ*ygaE*(pBAD*ygaE*) strains in hyperosmotic LB medium. Data are the mean ± SD from three independent experiments. (a) Expression of *ompF* in the Δ*ygaE* strain was increased threefold, compared to the wild-type strain. The complementation of *ygaE* to Δ*ygaE* strain repressed the expression of *ompF* to wild-type strain level. The expression of *ompF* in Δ*ygaE*(pBAD) was similar to Δ*ygaE *strain. (b) Expression of *ompC* in the Δ*ygaE *strain was increased fourfold, compared to the wild-type strain. The complementation of *ygaE* to Δ*ygaE* strain repressed the expression of *ompC* to wild-type strain level. The expression of *ompC* in Δ*ygaE*(pBAD) was similar to Δ*ygaE* strain.

Interestingly, our microarray results indicated that, compared to wild-type strain, the expression of *ompC* and *ompF* are obviously upregulated in Δ*ygaE* mutant strain under hyperosmotic stress and the expression of *ompC* is slightly more abundant than *ompF* in Δ*ygaE *strain (Table 3). The results of RT-PCR validated it; the expressions of *ompC* and *ompF* were increased fourfold, threefold, respectively, in Δ*ygaE* strain (Figures 2(a) and 2(b)), compared to wild-type strain after exposure to hyperosmotic stress 30 min. The completion of *ygaE* in Δ*ygaE* strain repressed the expression of *ompC* and *ompF* to wild type level (Figures 2(a) and 2(b)). The expression of the two genes in the strain which contained pBAD as control was similar to Δ*ygaE *strain (Figures 2(a) and 2(b)). These results suggested that *ygaE* is a repressor of *ompC* and *ompF*. Apparently, it is beneficial to decrease the influx channels when the osmotic stress is high in the environment, which will help the bacteria survival.

In *S*. Typhi, OmpC is always more abundant than OmpF, regardless of the growth conditions [16]. OmpC and OmpF are regulated by the OmpR and EnvZ proteins in *S*. Typhi, as in *E. coli* [16]. On the other hand, deletion of either *ompC* or *ompF* had no effect on expression of the gene coding for the other major porin: osmoregulation of OmpF synthesis was independent of OmpC expression; likewise, OmpC was still highly expressed in a Δ*ompF* background [16]. There appear to be unknown factors in *S*. Typhi that, together with the EnvZ and OmpR regulatory proteins, determine the particular behavior of OmpC expression [16]. Here we report that YgaE is a repressor of *ompC* in *S*. Typhi; it can be partially explained why the expression of *ompC* is not up-regulated under hyperosmotic stress. As for YgaE also repressing the expression of *ompF* in *S*. Typhi, we speculated that, in the evolution, the bacteria prefer to minus the influx pathways as more as possible to ensure the stability of the inner environment under hyperosmotic condition. The regulation of YgaE to *ompC* seems more obvious than *ompF*, which may due to the more abundant expression of *ompC* than *ompF*. However, whether the regulation of YgaE to *ompC* and *ompF* is direct and the concrete regulation mechanism still need further experiments to explore.

### 3.3. YgaE Does Not Affluence Growth and Antibiotic Susceptibility under Hyperosmotic Stress

Porin proteins control the permeability of polar solutes across the outer membrane of *E. coli* [35]. Optimal nutrient access is favored by larger porin channels as in OmpF protein [36]. But high outer membrane permeability is a liability in less favorable circumstances, and access of toxic agents or detergents needs to be minimized through environmental control of outer membrane porosity and the increased proportion of smaller OmpC channels in the outer membrane [37]. OmpF and OmpC of *E. coli* affect antibiotic transport and strain susceptibility [38–40]. Recent simulations pinpointed the specific interactions between antibiotics and key residues in the porin channels [41, 42].

**Figure 3:**
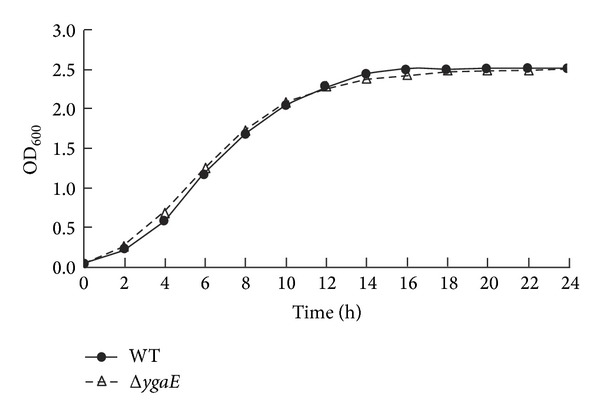
Growth of wild type (WT) and Δ*ygaE* in LB broth under hyperosmotic stress was monitored at OD_600_. Each datapoint represents the mean of three independent measurements.

**Figure 4:**
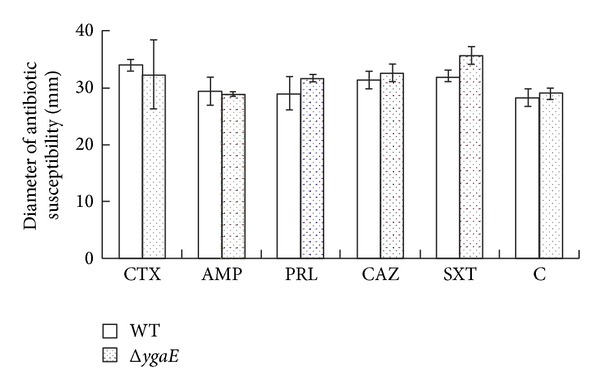
Antibiotic susceptibility of wild type and Δ*ygaE* under hyperosmotic stress measured by modified Kirby-Bauer disk diffusion method on Muller Hinton agar with a final NaCl concentration of 300 mM. The antibiotic disks which were used in this study were cefotaxime (CTX), ampicillin (AMP), piperacillin (PRL), ceftazidime (CAZ), compound sulfamethoxazole (SXT), and chloramphenicol (C). The zone size around each antimicrobial disk was measured. The experiment was performed three times. Student's *t*-test was used for the statistical analysis. Differences were considered statistically significant when *P* was < 0.05.

The repression of YgaE to *ompC* and *ompF* means less pathways for nutrition and antibiotics. To investigate whether the repression influences the nutrition influx under hyperosmotic stress in *S*. Typhi, we measured the growth of wild-type strain and Δ*ygaE *mutant in LB medium with a final NaCl concentration of 300 mM. Our results showed that the overall growth of both was similar, though the growth curve of Δ*ygaE *mutant seemed to be slightly higher than wild-type strain during the first ten hours (Figure 3); the differences were of no statistic meaning (*P* > 0.05). This result suggested that YgaE does not influence the growth of *S*. Typhi by the regulation of *ompC* and *ompF*. Next, in order to investigate whether YgaE affects the influx of antibiotics under hyperosmotic stress in *S*. Typhi, we examined the antibiotic susceptibility of wild-type and Δ*ygaE* strain to cefotaxime (CTX), ampicillin(AMP), piperacillin(PRL), ceftazidime(CAZ), compound sulfamethoxazole (SXT), and chloroamphenicol (C) by modified Kirby-Bauer disk diffusion method on Muller Hinton agar (OXOID) with a final NaCl concentration of 300 mM. The results displayed that the susceptibility of both strains to these antibiotics had no obvious differences (Figure 4).

One explanation for these phenomena is the repression of YgaE to *ompC* and *ompF* occurs only in the very early stage of hyperosmotic stress as an emergency approach to protect the bacteria. As time goes by, other mechanisms are involved in the process of handling the hyperosmotic stress, the repression of YgaE to *ompC* and *ompF* relieves. Another possibility is that the expression changes of *ompC* and *ompF *on transcription level do not lead to obvious decrease in OmpC and OmpF amount, which causes the unchanging phenotypes. All these speculations still needs more experiments to clarify.

### 3.4. YgaE Represses the Expression of OmpA at the Late Stage of Hyperosmotic Stress

**Figure 5:**
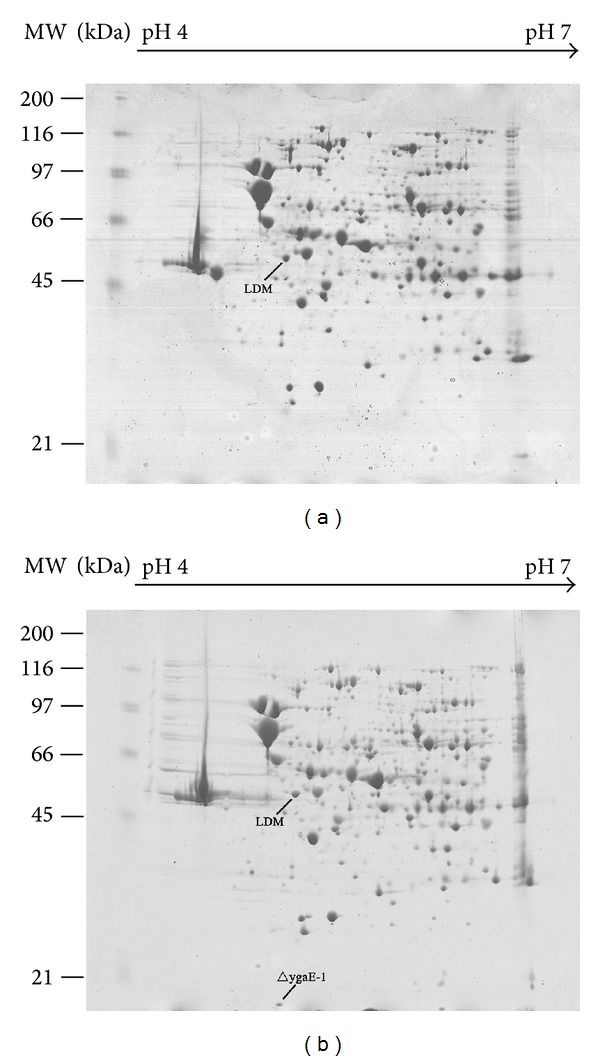
Two-dimensional electrophoresis was performed to compare the profiles of bacterial total proteins in wild-type strain and Δ*ygaE *strains after 120 min hyperosmotic shock. Protein spots that were unique to each strain were chosen for mass spectroscopy (MS) analysis. Δ*ygaE*-1 spot, which was discussed in this study, was unique to Δ*ygaE* strain and determined by MS to be the out membrane protein OmpA.

For revealing proteins probably regulated by YgaE in *S*. Typhi, a comparative proteomics approach was used to distinguish between the two-dimensional electrophoresis profiles of bacterial total proteins in wild-type strain and Δ*ygaE *strain. The total proteins of the two strains were obtained after 120 min stress growing in hyperosmotic LB culture and were analyzed in the pH range of 4–7. Protein spots that were unique to each strain were chosen for mass spectroscopy (MS) analysis. The MS analysis revealed that one of the unique proteins of Δ*ygaE *strain was identified to be OmpA (Figure 5), which indicated that YgaE represses the expression of OmpA at the late stage of hyperosmotic stress in *S*. Typhi.

OmpA is a key regulator of bacterial osmotic homeostasis modulating the permeability and integrity of the outer membrane in *E. coli* [43]. The predicted sequences of* S*. Typhi and *E. coli* OmpA proteins are nearly (>90%) identical [44]. In *S*. Typhi, OmpA is crucial for maintaining envelope integrity and preventing hemolysis through MV secretion [44]. OmpA can exist in two different conformations; a small part of this protein functions as a porin and the major part functions as an important structural protein [18]. Our microarray results revealed that the expression of *ompA* was similar in wild-type strain and Δ*ygaE *strain at the early stage of hyperosmotic stress. However, the 2-DE results showed that YgaE is a repressor of OmpA at the late stage of hyperosmotic stress. Oppositely, the expressions of *ompC* and *ompF* were obviously repressed by YgaE at the early stage and no apparent regulation of OmpC and OmpF by YgaE was observed in the 2-DE results at the late stage of hyperosmotic stress. These results together suggested that YgaE regulates out membrane proteins in a time-dependent manner under hyperosmotic stress in *S*. Typhi. The meaning of this regulation model lies in the fact that once the bacteria suddenly transfer to hyperosmotic environment, YgaE responses immediately to minus the influx channels to maintain the stability inside. Gradually, the bacteria adjust themselves and adapt to the environment, YgaE no longer tightly represses the expression of the two major porins OmpC and OmpF but transfers to repress the relatively less important porin OmpA, which also contributes to inner stable state of bacteria.

## 4. Conclusion

In the lumen of the host intestine, *S*. Typhi cells are exposed to a significant increase in osmolarity. The bacterial responses to hyperosmotic stress are complex. Our previous work found that RpoE and RpoS are two important sigma factors response to hyperosmotic stress and there are compensation and crosstalk between them [29]. YgaE is coregulated by RpoE and RpoS under hyperosmotic stress [29], which indicated its important role in *S*. Typhi under hyperosmotic stress. In this study, we firstly report that other than a repressor of *gab* operon in *S*. Typhi, YgaE also represses the expression of out membrane proteins. The repression of OMPs by YgaE is executed in a time-dependent manner: OmpC and OmpF are repressed in the early stage and OmpA is repressed in the late stage. We speculate that the reason of regulation pattern transformation of YgaE may be due to the expression variation of RpoE and RpoS: in the early stage of hyperosmotic stress, the decrease of RpoE and increase of RpoS [29] stimulate the expression of YgaE, and the accumulated YgaE represses the expression of OmpF and OmpC. In the late stage of hyperosmotic stress, RpoE and RpoS reached a balanced level [29]; once YgaE senses the balance, it transfers to repress the expression of OmpA.

This study provides new insight into the regulation of out membrane proteins under hyperosmotic stress in *S*. Typhi, which will help us to better understand the adaptation of *S*. Typhi to hyperosmotic shock once invading the host.

## Figures and Tables

**Table 1:** Strains and plasmids used in this study.

Strains or plasmid	Relevant characteristics	Source
Strains		
*Salmonella enterica *serovar Typhi GIFU10007	Wild-type strain; z66^+^	Gifu University [22]
Δ*ygaE *	GIFU10007; z66^+^	This study
Δ*ygaE* (pBAD)	Δ*ygaE* harboring pBAD plasmid;	This study
Δ*ygaE* (pBAD*ygaE*)	Δ*ygaE* harboring pBAD-*ygaE* recombinant plasmid;	This study
* E. coli* SY372*λ*pir	*E. coli* host strain of suicide plasmid	Gifu University [23]
Plasmids		
pGMB151	Suicide plasmid; sacB; Amp^r^	Gifu University [23]
pBAD/gIII	Expression vector; Amp^r^	Invitrogen
pBAD*ygaE *	pBAD/gIII containing *ygaE* gene	This study

**Table 2:** Primers used in this study.

Name	Sequence
*ygaE*-F1A(+BamH I)	5′-TTGGATCCGGTACTGTCCCCATTATGT
*ygaE*-F1B(+Sal I)	5′-CCGTCGACATAGCCTTTTTGATTCACC
*ygaE*-F2A(+Sal I)	5′-TAGTCGACGAGATGCTGGAAGATAAAC
*ygaE*-F2B(+BamH I)	5′-CTGGATCCTCTGCTACACTTTTCTTTG
*ygaE*-PA(+Nco I)	5′-AACCATGGAGATGACCGCCCTTTCCCAA
*ygaE*-PB(+Sal I)	5′-AAGTCGACCTACATTTTTCCTGCCAT
P-*ompF*-A	5′-GGA ATACCGTACTAA AGCA
P-*ompF*-B	5′-GATACTGGATACCGA AAGA
P-*ompC*-A	5′-ATCAGA ACAACACCGCTAA
P-*ompC*-A	5′-GTTGCTGATGTCCTTACC

**Table 3:** Gene expression changes in Δ*ygaE *under hyperosmotic stress discussed in this study.

Gene name	Description of gene product	log_2_(Δ*ygaE/*WT)
*gab* operon genes		
* ygaF *	Putative GAB DTP gene cluster repressor	1.68
* gabD *	Succinate-semialdehyde dehydrogenase	1.44
* gabT *	4-Aminobutyrate aminotransferase	1.65
* ygaE *	Putative transcriptional regulator	−3.62
Outer membrane protein genes		
* ompC *	Out membrane protein C	1.76
* ompF *	Outer membrane protein F precursor	1.37
